# Supplementary figures and images for: Genome-wide characterization and expression analysis of soybean trihelix gene family
Source: PeerJ. 2020 Mar 13;8:e8753. doi: 10.7717/peerj.8753 (PMC7075366; doi:10.7717/peerj.8753)

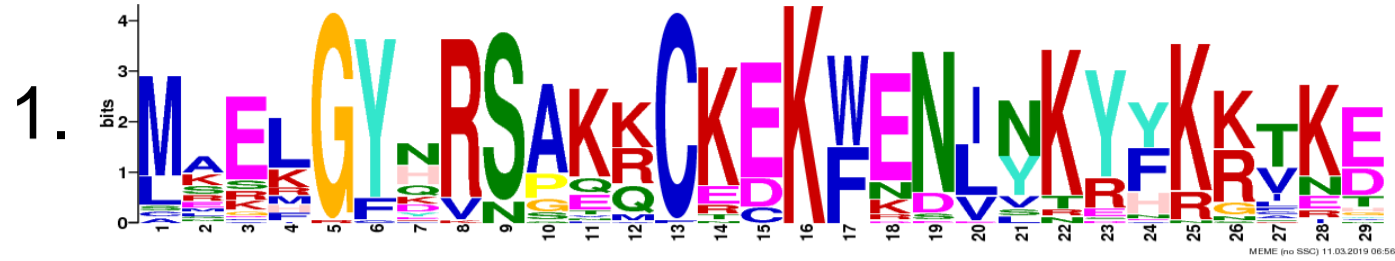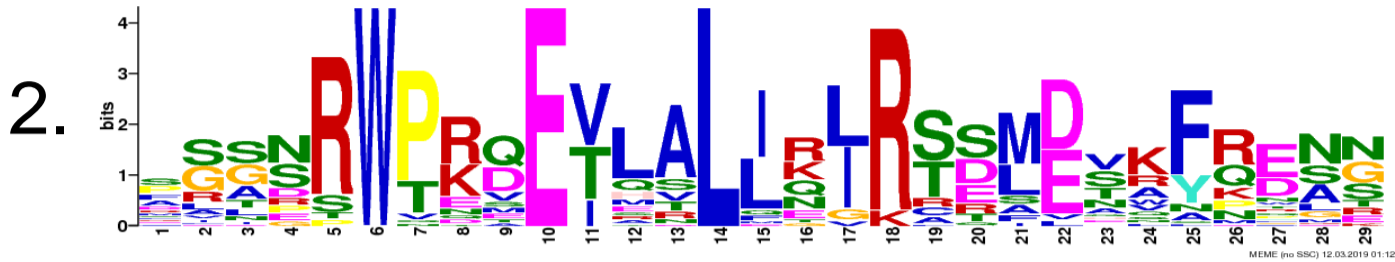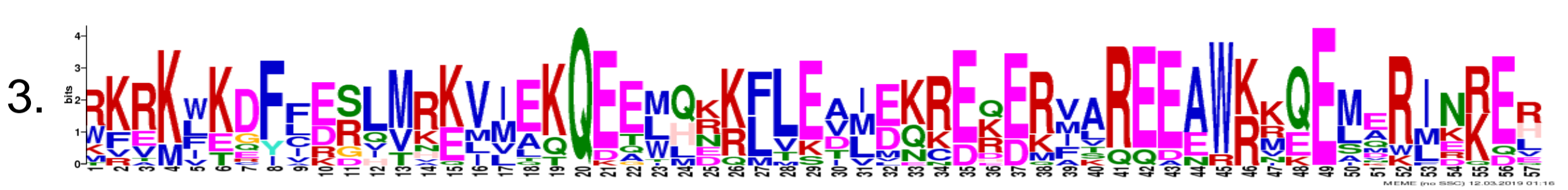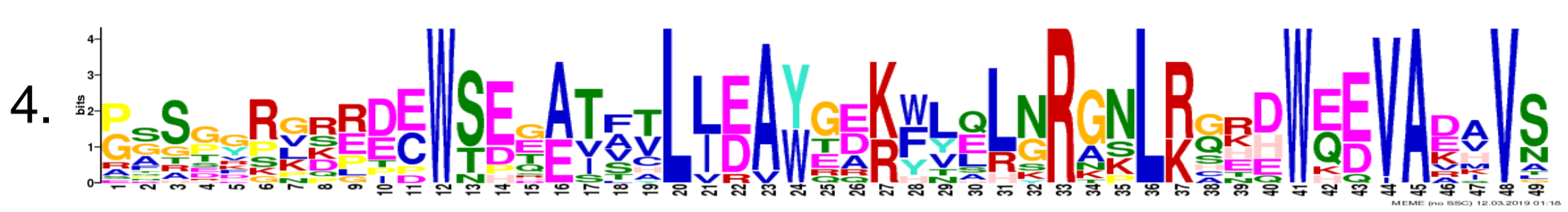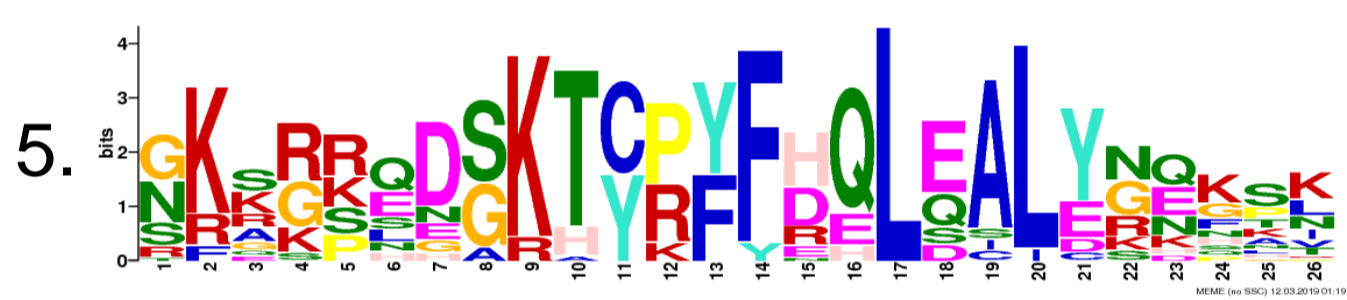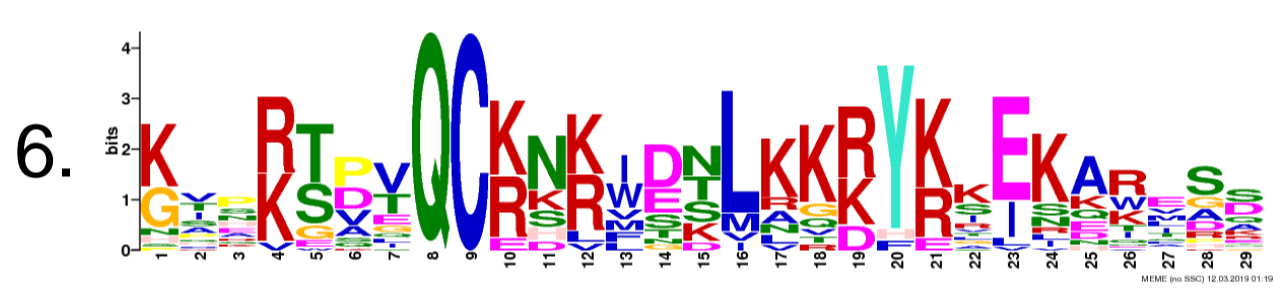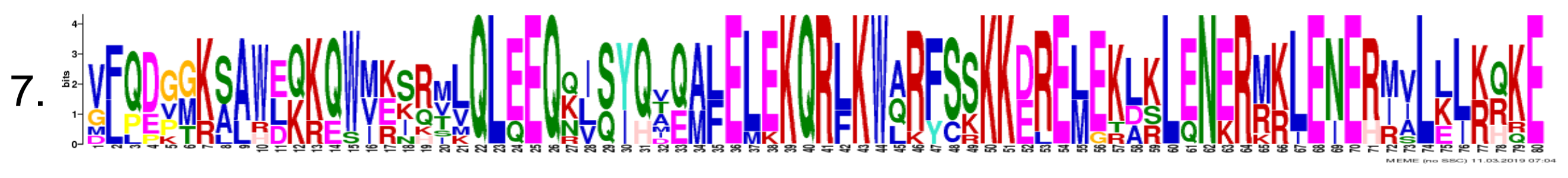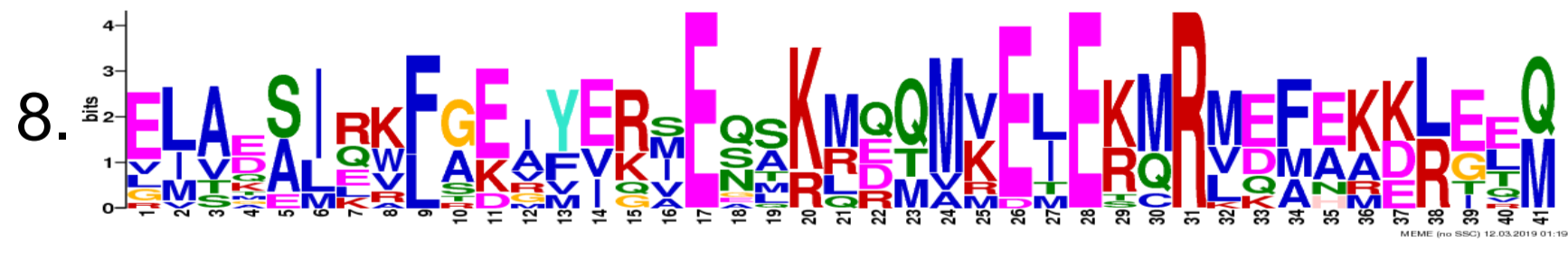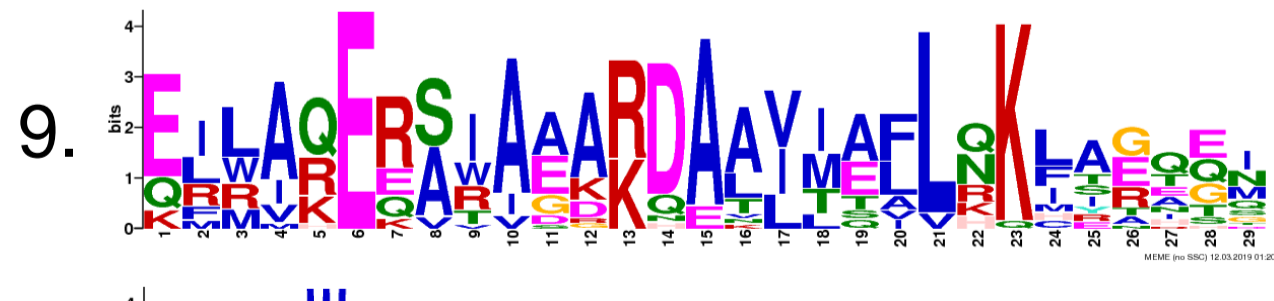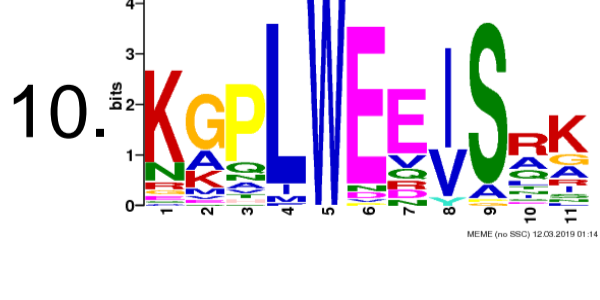

Supplement: Figure S2 [file peerj-08-8753-s002.pdf]

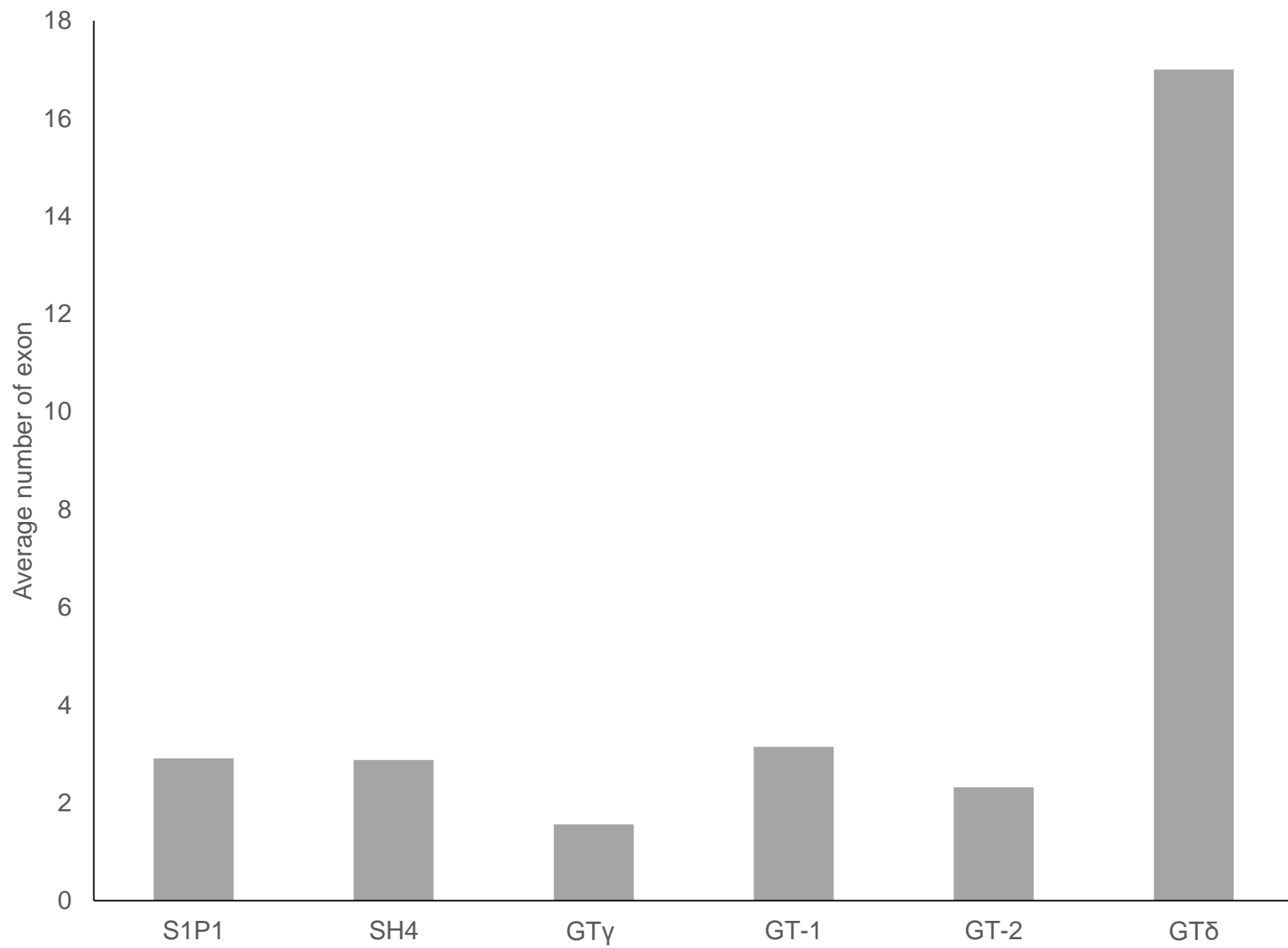

Supplement: Figure S3 [file peerj-08-8753-s003.pdf]

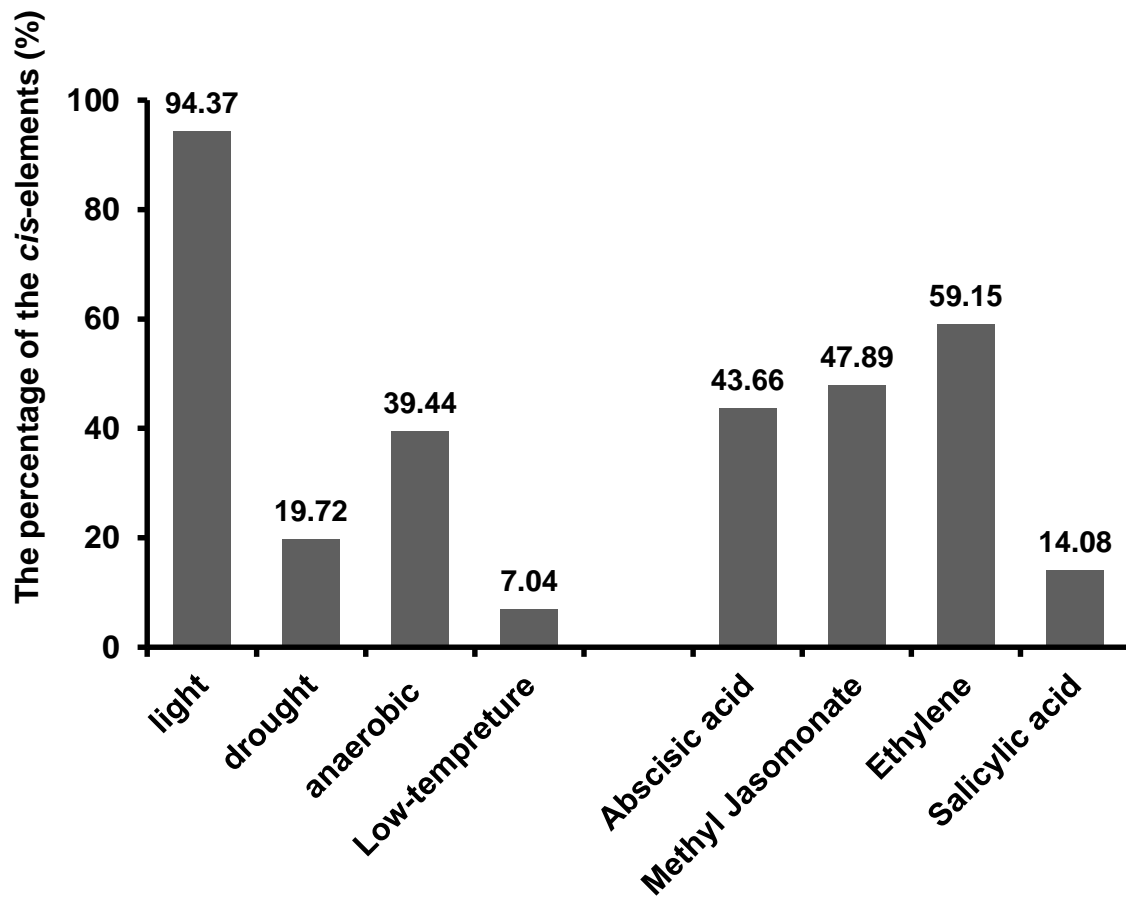

Supplement: Figure S4 [file peerj-08-8753-s004.pdf]

Relative expression

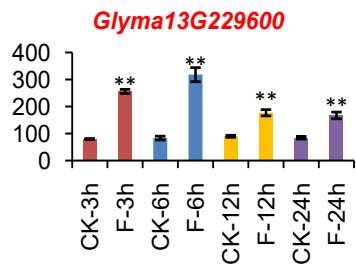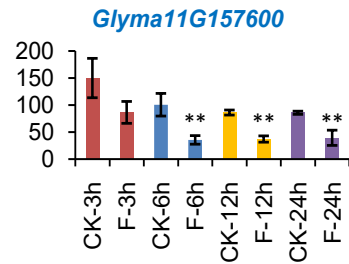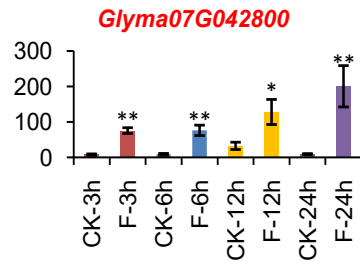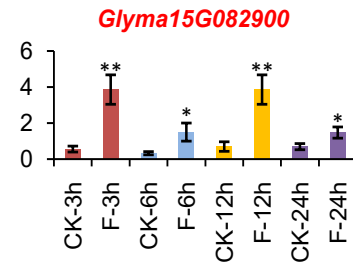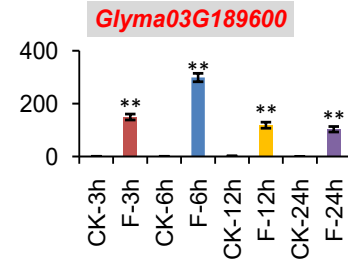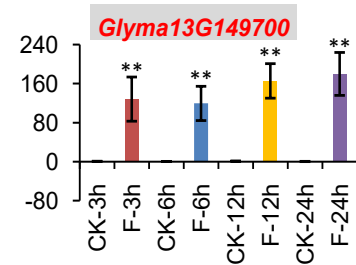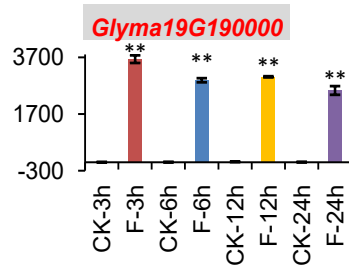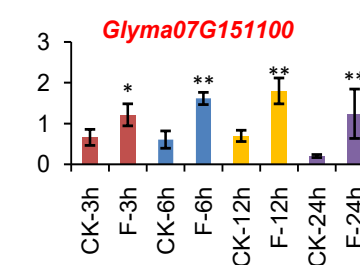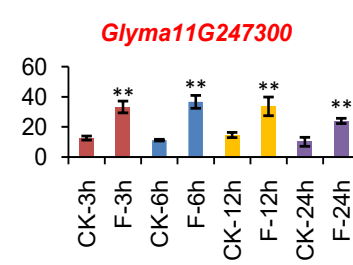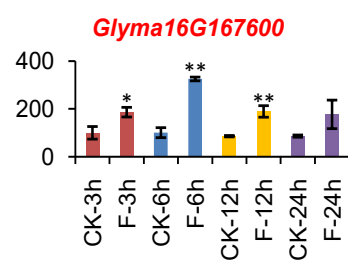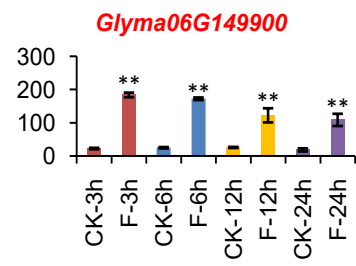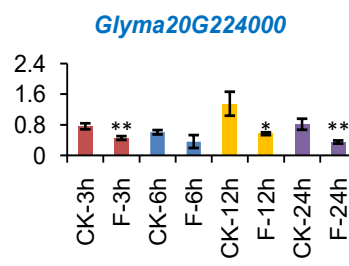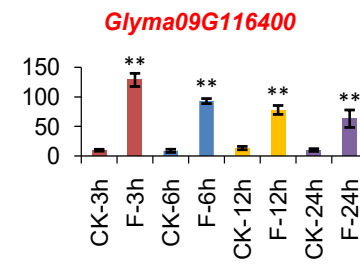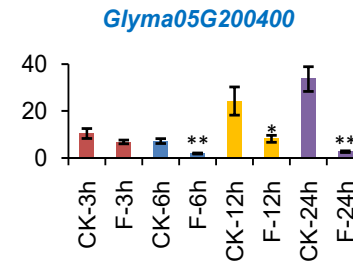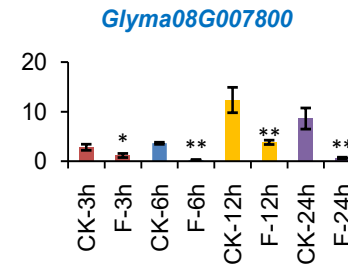

Supplement: Figure S5 [file peerj-08-8753-s005.pdf]
