# Supplementary material for: Genome-wide characterization and expression analysis of soybean trihelix gene family
Source: PeerJ. 2020 Mar 13;8:e8753. doi: 10.7717/peerj.8753 (PMC7075366; doi:10.7717/peerj.8753)
Supplement: Figure S6 [file peerj-08-8753-s006.pdf]

		root	root hairs	nodules	stem	SAM	leaves	flower	pod	seed
Glyma01G121000 vs Glyma03G056900	*Glyma01G121000*	0.160619	0.248755	0.618115	0.277469	0.023313	0.851996	0.226749	0	0
	Glyma03G056900	0.444181	0.610239	1.36029	1.31391	0.075527	0.83417	2.35939	0.217123	0.092367
Glyma11G247300 vs Glyma18G010000	*Glyma11G247300*	7.87504	6.28703	8.28761	6.324	7.51652	7.66629	10.3313	7.98244	11.939
	Glyma18G010000	5.73215	4.83253	4.37537	4.96315	7.01261	6.12043	7.72388	5.35083	7.20971
Glyma04G216100 vs Glyma09G116400	*Glyma04G216100*	2.01793	1.34586	1.48033	1.23753	2.30618	8.2547	4.72849	1.95722	6.19918
	Glyma09G116400	1.83779	1.39403	1.44248	2.53842	3.84133	13.6896	3.55846	4.84984	7.16425
Glyma09G116400 vs Glyma06G149900	*Glyma09G116400*	1.83779	1.39403	1.44248	2.53842	3.84133	13.6896	3.55846	4.84984	7.16425
	Glyma06G149900	8.90306	2.20722	2.82885	1.81401	2.22461	5.98026	7.89562	0.562422	1.10702
Glyma10G202300 vs Glyma20G188100	*Glyma10G202300*	1.01876	0.766811	2.38298	10.766	4.49362	0.028172	0.293803	7.19561	0.307679
	Glyma20G188100	0.741852	0.480506	1.4559	3.01268	2.34651	0	0.039315	0.208075	0.179956
Glyma19G190000 vs Glyma03G189600	*Glyma19G190000*	0.771785	2.94245	6.20492	1.34016	2.05404	2.97999	0.290096	0.564081	1.7541
	Glyma03G189600	0.444625	15.7617	49.7626	0.0524641	0.026414	0.94922	0	0.367246	12.7768
Glyma04G194600 vs Glyma06G171400	*Glyma04G194600*	2.83884	2.09338	1.80004	3.06228	3.84802	7.72941	10.6828	3.52061	3.65722
	Glyma06G171400	1.84175	3.46008	1.8496	5.87014	11.3159	14.4378	4.88157	5.85753	2.67731
Glyma10G065100 vs Glyma13G149900	*Glyma10G065100*	2.46982	3.88209	4.03298	3.57722	3.89898	3.68247	3.41384	3.64869	4.30375
	Glyma13G149900	2.70582	4.16189	4.89908	4.48978	4.4701	6.43813	5.0527	5.89147	5.41986
Glyma12G210900 vs Glyma13G290500	*Glyma12G210900*	2.07574	2.24192	2.37543	1.47902	2.01798	1.10835	1.76709	2.43741	1.6352
	Glyma13G290500	1.77946	2.1994	2.17884	2.11521	1.43689	2.66671	2.09713	1.90186	1.57953
Glyma13G195800 vs Glyma15G234100	*Glyma13G195800*	5.67958	6.23178	8.13482	10.6329	5.45297	3.23444	5.60333	9.50166	3.77473
	Glyma15G234100	5.83403	5.08273	12.9437	12.2993	4.19154	1.29944	2.67017	10.418	7.83963
Glyma13G359800 vs Glyma15G014200	*Glyma13G359800*	2.75258	1.76078	3.21281	4.18568	3.81405	4.98121	2.2326	3.06394	3.73436
	Glyma15G014200	3.15148	2.13419	3.55901	3.77923	3.02418	3.87305	2.81221	1.8372	2.48522
Glyma10G298700 vs Glyma20G249900	*Glyma10G298700*	4.88493	12.7034	36.3941	3.46082	3.08699	4.30002	3.07952	3.11447	5.73533
	Glyma20G249900	6.10694	6.86154	13.3524	7.34687	3.9675	6.42809	4.00223	5.69598	5.87891
Glyma08G257500 vs Glyma18G281800	*Glyma08G257500*	3.12346	4.41717	9.86534	4.28739	1.91305	2.35747	2.1459	5.72506	5.46577
	Glyma18G281800	1.44784	2.57967	5.35819	1.74489	0.885066	1.59408	0.66809	3.36854	2.77625
